# Oligodeoxynucleotides ODN 2006 and M362 Exert Potent Adjuvant Effect through TLR-9/-6 Synergy to Exaggerate Mammaglobin-A Peptide Specific Cytotoxic CD8+T Lymphocyte Responses against Breast Cancer Cells

**DOI:** 10.3390/cancers11050672

**Published:** 2019-05-14

**Authors:** Duaa Babaer, Suneetha Amara, Brenda S. McAdory, Owen Johnson, Elbert L. Myles, Roy Zent, Jeffrey C. Rathmell, Venkataswarup Tiriveedhi

**Affiliations:** 1 Department of Biological Sciences, Tennessee State University, 3500 John A Merritt Blvd, Nashville, TN 37209, USA; duaa.a.b@hotmail.com (D.B.); bmcadory@tnstate.edu (B.S.M.); emyles@tnstate.edu (E.L.M.); 2 Department of Medicine, St Thomas Hospital-Midtown, Nashville, TN 37203, USA; suneetha.amara@ascension.org; 3 Department of Public Health, Health Administration & Health Sciences, Tennessee State University, Nashville, TN 37209, USA; ojohnson6@tnstate.edu; 4 Medicine, Cell & Developmental Biology, Vanderbilt University School of Medicine. Veterans Affairs Medical Center, Nashville, TN 37212, USA; roy.zent@vumc.org; 5 Department of Pathology, Microbiology, and Immunology, Vanderbilt University Medical Center, Nashville, TN 37212, USA; jeff.rathmell@vumc.org; 6 Department of Pharmacology, Vanderbilt University, Nashville, TN 37212, USA

**Keywords:** breast cancer, mammaglobin-A, cancer vaccine, adjuvant, synthetic oligonucleotide, toll-like receptor (TLR)

## Abstract

Mammaglobin-A (MamA) is overexpressed in 40–80% of all human breast cancers. Recent phase I clinical trials of the MamA DNA vaccine showed encouraging safety outcomes. However, this vaccine elicited only a modest increase in MamA specific CD8+T lymphocyte (CTL) activation. As vaccine adjuvants play a critical role in enhancing the immunotherapeutic efficiency of vaccines, we tested the potential role of three synthetic CpG oligodeoxynucleotides (ODN2216—class A ODN, ODN2006—class B ODN, and ODN M362—class C ODN) to further enhance MamA specific CTL responses. Towards this, naïve CD8+T cells were obtained from healthy HLA-A2+ human donors. The HLA-A2 specific immunodominant epitope of MamA, MamA2.1 (LIYDSSLCDL), was utilized to activate naïve CD8+T cells. The THP-1 (HLA-A2+) cells were used as antigen presenting cells to stimulate naïve CD8+T cells along with (or without) co-treatment of various ODNs mentioned above. Activation of naïve CD8+T cells with the MamA2.1 peptide along with ODNs demonstrated enhanced MamA specific CTL mediated cytotoxicity on AU565 (HLA-A^+^/MamA^+^) breast cancer cells following co-treatment with ODN2006 and M362 compared to ODN2216 or MamA2.1 peptide alone. However, no significant cytotoxicity was noted upon treatment of MamA2.1 activated CTLs on MCF7 (HLA-A^+^/MamA^−^) cells, suggesting that the activation of CTLs is specific to the MamA antigen. Functional characterization studies demonstrated specific IL-12 mediated cross-talk between TLR-6 and -9 in THP-1 cells following stimulation with ODN2006 and M362, which was critical for the final cytotoxic activation of CD8+T lymphocytes. Based on these data, we conclude that ODN2006 and ODN M362 exerted a strong adjuvant effect through induction of the initial innate immune response through TLR9 upregulation followed by enhanced MamA specific CTL dependent adaptive immune responses. Our current data provide evidence for the application of Class-B/-C-CpG-ODNs as potential vaccine adjuvants towards enhancing the success of MamA based breast cancer vaccination.

## 1. Introduction

Cancer facts and figures (2018) estimate that breast cancer accounts for 15.5% of all newly diagnosed cancers in the United States [1]. Advances in the understanding of molecular biology and immunology provide an important futuristic application of DNA and peptide-based vaccines as viable long-term anti-cancer immunotherapeutic strategies. Recent availability of relatively low-cost recombinant plasmid development tools and high-throughput strategies to efficiently identify novel tumor associated antigens has significantly reduced the cost and the time involved in DNA vaccine preparation [2]. The long-lasting therapeutic success of a DNA vaccine depends upon the ability of the transfected gene coding for specific tumor associated antigens (TAA) in the plasmid backbone to efficiently activate antigen specific cytotoxic and memory CD8+T lymphocytes [3]. We recently proposed a “five-force immune hypothesis” to identify and develop various strategies towards the success of anti-cancer vaccine therapy [4]. As we previously noted, vaccine adjuvants play a critical role by acting as “indirect activators” to supplement immunogenicity and efficiency of DNA vaccines.

Mammaglobin-A (MamA) is a human breast cancer-associated antigen expressed in 40–80% of primary and metastatic breast cancers, thus making MamA a potential antigen to develop breast cancer specific vaccines [5,6,7,8,9]. The levels of MamA expression are comparable among well-differentiated, moderately differentiated, and poorly differentiated breast tumors [7]. Although MamA is extensively expressed in human breast tumors, none of the established murine breast cancer cells express MamA. Therefore, an absence of MamA breast cancer murine ortholog limits small animal based MamA antigenic characterization studies. However, previous partial murine based studies by Narayanan et al. demonstrated that the passive transfer of T cells from human MamA DNA sequence vaccinated F1-HLA-A2/hCD8 double transgenic mice into a NOD/SCID (non-obese diabetic/severe combined immunodeficient) mouse with orthotopically implanted human breast cancer cells resulted in a significant reduction of tumor size in an HLA-A2 and a MamA specific manner [10]. Furthermore, it was determined that MamA2.1 (amino acids 83-92, LIYDSSLCDL) exerted HLA-A2 specific immunodominance. Importantly, both transgenic murine and human breast cancer studies performed in the context of HLA-A2 antigen presentation have shown that there is an expansion of MamA2.1 specific cytotoxic CD8+T-cells, thus suggesting that MamA2.1 is the immunodominant epitope to stimulate effector CD8+T cell responses [10,11]. Based on these results, a phase I clinical trial of the MamA cDNA vaccination was initiated in stage-IV breast cancer patients [12,13]. Results from this study demonstrated that MamA vaccinated breast cancer patients had significantly improved progression-free survival (PFS) and enhanced expansion of MamA2.1+CD8+T lymphocytes. In this study, it was important to note that, following MamA-cDNA vaccination, there was only a modest increase in the frequency of MamA2.1+CD8 T cells. Addition of an appropriate vaccine adjuvant could act as an “indirect activator” to further enhance the effector CD8+T cell responses following MamA vaccination.

One of the important prerequisites for successful outcomes following human peptide or DNA-based vaccination strategy is the correct choice of a vaccine adjuvant [4]. Synthetic oligodeoxynucleotides (ODNs) containing unmethylated CpG motifs have been extensively studied for their roles as efficient vaccine adjuvants [14]. These adjuvants work as ligands and bind to innate immune activating toll-like receptors (TLR), leading to a stimulation of downstream inflammatory cytokine (such as interferon cytokine family) signaling pathway [15]. In the context of vaccination, the TLR stimulation initiates innate immune responses at the site of vaccine injection and provides the first line of active defense, along with enhancing the phagocytic efficiency and the eventual antigen presentation by profession antigen presenting cells, such as macrophages and dendritic cells. This will subsequently mount an effective adaptive immune response characterized by the expansion of antigen (Ag)-specific CD8+T lymphocyte (CTL) response. While unmethylated CpG ODNs are present at a high frequency in bacterial DNA, they are extremely rare in eukaryotic DNA. Bacterial DNA released during active infections induces TLR9 expression and subsequent downstream signaling, culminating in the elimination of the pathogen [16]. Interestingly, TLR9 knock-out based murine studies demonstrated that these mice could not mount a strong innate immune response to stimulation by ODNs, suggesting that TLR9 is a key molecule in inducing ODN mediated immunostimulatory response [17]. Furthermore, murine colon cancer studies by Mukerjee et al. have shown that the vaccination of MHC (major histocompatibility complex) class I restricted cell surface-associated mucin (MUC1) peptide combined with ODN elicited a stronger anti-tumor response compared with MUC1 peptide alone [18]. As mentioned above, in view of the limitations in performing full-fledged murine studies due to absence of murine breast cancer MamA ortholog, in this communication, we studied the impact of co-treatment of ODNs with the HLA-A2 restricted MamA peptide (MamA2.1 peptide) to characterize the potential activation and the effector capabilities of naïve CD8+T lymphocytes (CTLs) collected from HLA-A2+ healthy human subjects. 

## 2. Materials and Methods

### 2.1. Cell Lines and Healthy Human CD8+T Lymphocytes

The breast cancer cell lines, AU565 (MAM-A^+^/HLA-A2^+^) and MCF-7 (MAM-A^−^/HLA-A2^+^), and human monocyte-like cell line, THP-1 cells, were obtained from the American Type Culture Collection (ATCC, Manassas, VA, USA). Human CD8+T cells from HLA-A2^+^ healthy subjects were obtained from StemCell technologies (Cambridge, MA, USA). All cell cultures and incubations were performed as per provider’s recommendations and described by us before [19,20]. Briefly, cells were cultured in RPMI-1640 medium at 37 °C in a 5% CO_2_ incubator until they were 80% confluent. The presence of MamA and HLA-A2 expression in the breast cancer cell lines was confirmed by western blot analysis (data not shown). The ODNs—ODN2216, ODN2006, and ODN M362 (respectively controls represented by suffix ‘c’)—were at vaccigrade and obtained from InvivoGen (San Diego, CA, USA). For ODN stimulation, the breast cancer cells and the THP-1 cells were cultured in 24 well plates, 1 × 10^5^ per well pre-stimulated with PMA (phorbol myristate acetate, 10 ng/mL medium) for 16 h, washed once with RPMI-1640 media, and stimulated with ODNs (10 µM) for 5 h. These cells were later used for various experiments detailed below. 

### 2.2. Animal Studies Section

C57BL/6J mice carrying the HLA-A*02:01 gene (HLA-A2+) were obtained from Jackson Laboratories. SCID beige mice were purchased from Charles River Laboratories. Mice were housed in specific pathogen-free facilities in ventilated cages with at most 5 animals per cage and were provided ad libitum food and water. For vaccination, 100 µg/mice of endotoxin free MamA2.1 peptide (confirmed by Limulus amebocyte lysate [LAL] assay) was injected into the peritoneal cavity with or without the three ODNs (100 µg/mice for a given ODN type) mentioned above. All protocols followed the guidelines approved by the Vanderbilt Institutional Animal Studies Committee. This article does not contain any studies with human participants or animals performed by any of the authors. The primary human immune cells utilized for study in this article were purchased from a commercial source (StemCell Technologies, Cambridge, MA, USA).

### 2.3. Immunogenic Peptide Stimulation

For MamA2.1 peptide stimulation (LIYDSSLCDL), CD8+T lymphocytes (1 × 10^6^) were cultured in 2 mL of supplemented RPMI-1640 media in 24-well plates in the presence of irradiated (5000 rads) THP-1 cells (1 × 10^6^) loaded with MamA2.1 in the presence of β2m (3 µg/mL), CD3 (500 ng/mL), CD28 mAb (500 ng/mL), and recombinant human IL-2 (20 U/mL), then used for efficient antigen presentation to naïve CD8+T cells (0.5 × 10^5^ cells maintained at 20:1 ratio of THP-1 to CD8+T cells) collected from healthy HLA-A2^+^ donors (StemCell Technologies, Cambridge, MA, USA). The MamA and the peptide specific cytotoxic activities of the resulting CD8+ CTL lines were analyzed 9 days after 3 stimulations. At the end of 3 stimulations, CD8+T lymphocytes were isolated by immunomagnetic separation (MACS Miltenyi Biotec, San Diego, CA, USA), and the resulting purity was verified to be >95%. For specific TLR-9 knock down in AU565 cells by siRNA, we utilized TLR9-siRNA sense sequence: CCGCAUCGUCAAACUGGCGtt, antisense sequence: CGCCUGTTTGUCGUTGCGGtt, and for scramble control, CCCAUUCGGGGGCAAACUCtt was utilized [21,22]. For TLR-6 knock down, the following three siRNAs were used: TLR6 siRNA-1 sense sequence: CCAGAAUCCAGUUCUCCGAtt, antisense sequence: UCGGAGAACUGGAUUCUGGtt; TLR6 siRNA-2 sense: GCAACUUAUCACAACUGAAtt, antisense sequence: UUCAGUUGUGAUAAGUUGCca; TLR6 siRNA-3 sense sequence: GGUGCUUACAACUGACUAAtt, antisense sequence: UUAGUCAGUUGUAAGCACCct. The MamA2.1 peptide was synthesized by peptide 2.0 Inc. (Chantilly, VA, USA) and purified on HPLC column to >95% purity. All chemicals and reagents, unless otherwise mentioned, were obtained either from Sigma–Aldrich (St Louis, MO, USA) or Fisher Scientific (Hampton, NH, USA).

### 2.4. Cytotoxicity Assay

The cytotoxic efficiency of peptide-activated CD8+T cells was investigated by its ability to lyse the target breast cancer cells by non-irradiative LDH (lactate dehydrogenase) release assay (Promega, Madison, WI, USA) [12]. The breast cancer cells (1 × 10^4^ cells, referred to as target cells) incubated at 37 °C in 200 µL of complete medium were plated in quadruplicate cultures in round bottom 96-well plates in the presence of varying numbers of activated CD8+T cells (referred to as effector cells), with a effector to target (E:T) ratio maintained at 6.25:1 to 50:1. The percentage specific lysis was calculated as follows: [(experimental LDH release − spontaneous LDH release)/(maximum LDH release − spontaneous LDH release)] × 100.

### 2.5. Western Blot

Total proteins were extracted from cells with lysis buffer. The supernatant was collected after running at 13,500 rpm for 20 min at 4 °C, as previously described [23]. Protein concentration was determined with a Bradford assay kit from Bio-Rad (Philadelphia, PA, USA). Total proteins were separated on a 4–12% sodium dodecyl sulfate-polyacrylamide gradient gel and electrophoretically transferred onto a nitrocellulose membrane. The membranes were blocked overnight at 4 °C in Tris-buffered saline with 0.05% Tween 20 (5% nonfat milk in 10 mM Tris-HCl 100 mM NaCl 0.1% Tween 20, pH 7.4). The membranes were incubated first with antibodies (Abs) specific for total and phosphorylated forms at room temperature with primary Abs diluted 1 in 1000 in blocking buffer for 2 h and then with a horseradish peroxide-conjugated secondary IgG mAb diluted 1 in 5000 for 1 h. All primary and secondary Abs were obtained from Santa Cruz Biotech (Dallas, TX, USA) unless indicated otherwise. The following specific primary antibodies to TLR-9 (sc-52966), Perforin (sc-33655), and Actin (sc-10731) were utilized. The membrane was developed using the chemiluminescence kit (Millipore) and analyzed using Bio-Rad Universal Hood II (Hercules, CA, USA). Morphometric analysis was done using the software provided by the company.

### 2.6. Luminex

Whole cell lysates were prepared and quantified by BCA Protein Assay Kit (Pierce Biotechnology/Thermo Fisher Scientific, Rockford, IL, USA), and 250 μg of protein was equalized and used per sample in the cytokine magnetic beads (Bioplex Pro human cytokine 27-plex, BioRad, Herculus, CA, USA) as per the manufacturer’s reagents and protocol. The samples were analyzed on a BioPlex-200 instrument and data analyzed using BioPlex Manager^TM^ software. The median fluorescent intensity (MFI) data were obtained with a detection target of 50 beads per region, low RP1 target for CAL2 calibration, and recommended doublet discriminator (DD) gates of 5000–25,000. The data were analyzed by comparing standard, control, and sample wells, with bead counts <37 excluded, as this was the minimum number required to reduce the potential impact of outlier beads on MFI. We excluded from the standard curve any points with (%CV) less than 25%, and those with accuracy outside of 80–120% of the expected range were excluded, starting from the lowest standard. The analysis software was then used to fit a curve to this set of reliable standards data using five parameter logistic regression with default automated weighting fitted to at least 6 points. Protein values are presented as mean ± SD and * *p* < 0.05 (ANOVA, one-way) comparing the Mama2.1 peptide treatment alone. N.D. (non-detectable) indicates the protein detection was below the minimum detection range for that particular cytokine.

### 2.7. Enzyme Linked Immunosorbent Assay (ELISA)

The secretory extracellular interferon-γ (IFNγ) (R&D Systems, Minneapolis, MN, USA) and perforin (AbCam, Cambridge, MA, USA) in the cell supernatant was quantitated by ELISA as per the manufacturer’s protocol [24]. Given the limitation of the detection, the supernatant was diluted 1:1000 and quantified with a standard curve using the manufacturer provided standards. Detection at 450 nm was performed using EMax Plus spectrophotometer, and data analysis was carried out using software provided by the manufacturer (Molecular Devices, Sunyvale, CA, USA).

### 2.8. Quantitative Real Time Polymerase Chain Reaction (qRT-PCR)

Expression profiles of genes in the THP1 cells were analyzed using the FAM-labeled RT-PCR primers for TLR1 (Hs00413978_m1), TLR2 (Hs02621280_s1), TLR3 (Hs01551079_g1), TLR4 (Hs00152939_m1), TLR5 (Hs01920773_s1), TLR6 (Hs01039989_s1), TLR7 (Hs01933259_s1), TLR8 (Hs00152972_m1), TLR9 (Hs00370913_s1), TLR10 (Hs01935337_s1), Perforin (Hs00169473_m1), GADPH (Hs402869), and Actin (Hs4333762T), and for murine genes TLR6 (Mm02529782_s1) and TLR9 (Mm00446193_m1) obtained from Applied Biosystems/Life Technologies (Grand Island, NY, USA) as per the manufacturer’s recommendation [22]. Briefly, total RNA was extracted from 10^6^ cells using TRIzol reagent (Sigma–Aldrich, St Louis, MO, USA). RNA samples were quantified by absorbance at 260 nm. The RNA was reverse-transcribed, and RT-PCR was performed in a final reaction volume of 20 μL using iCycler 480 Probes Master (Roche Diagnostics, Indianapolis, IN, USA). Each sample was analyzed in triplicate. Cycling conditions consisted of an initial denaturation of 95 °C for 15 min, followed by 40 cycles of 95 °C for 30 s, followed by 61 °C for 1 min.

### 2.9. Flow Cytometry

The intracellular TLR-9 expression in cells was analyzed by flow cytometry using appropriate fluorophore-labelled primary antibodies (BioLegend, SanDiego, CA, USA) [22,25]. MamA2.1 tetramers were developed by Beckman Coulter Immunomics (San Diego, CA, USA) to monitor the MamA-specific CD8+T cell response following MamA DNA vaccination. An HLA-A2 tetramer incorporating an unrelated peptide from influenza (Flu), GILGFVFTL, was also prepared and used as a control. Tetramers were used to stain target cells at a concentration of 10 µL per 200 µL with a final volume of CD8+T cells (1 × 10^6^ CD8+T cells/mL). Antibodies used for flow cytometry included CD8-FITC (BD Biosciences, San Jose, CA, USA), MamA2.1/Tetramer-PE (phycoerythrin), and Flu-peptide/Tetramer-PE. Samples were analyzed using a FACS Calibur^TM^/LSRII flow cytometer (Becton-Dickinson, Franklin Lakes, NJ, USA), and cell sorting was performed using a Vantage cell sorter (Becton-Dickinson). Data were analyzed using BD FACSDiva software (BD Biosciences, San Jose, CA, USA). Gates were set according to isotype controls. 

### 2.10. Statistical Analysis

Data are expressed as mean ± SD from four independent studies. Statistical differences between means were analyzed using a paired or unpaired Student’s *t* test. A *p*-value of less than 0.05 was considered significant. All data analyses were obtained using Origin 7 software (Origin Labs, Northampton, MA, USA) or GraphPad5 (Graph Pad Software, LaJolla, CA, USA).

## 3. Results

### 3.1. Enhanced MamA2.1 Specific CD8+T-Cell Mediated Breast Cancer Cell Cytotoxicity following Treatment with ODN2006 and M362

**Figure 1:**
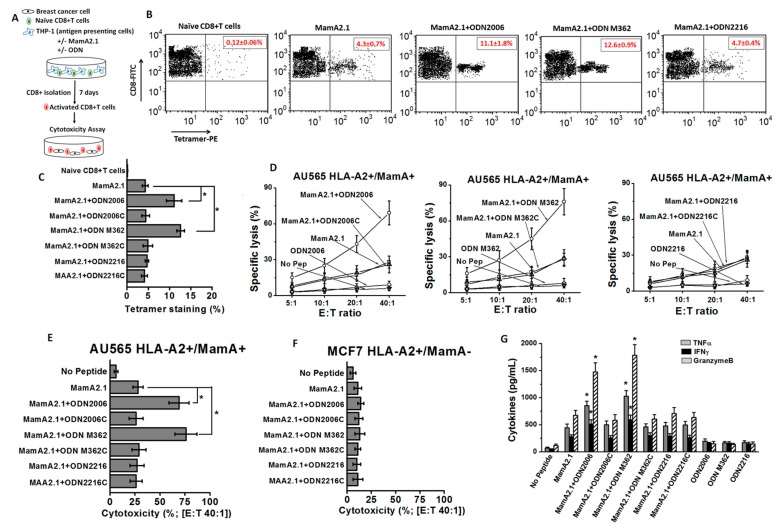
Activation of CD8+T lymphocytes (CTLs) following antigen presentation by THP1 cells and stimulation by MamA2.1 peptide with and without ODNs. (**A**) Schematic of the experimental design. (**B**,**C**) MamA2.1 tetramer staining of the CTLs. The naïve CD8+T cells were stimulated by THP-1 cells pre-treated with either the MamA2.1 peptide alone or in combination with various ODNs. (**D**) Cytotoxicity of CTLs activated under various treatment conditions (mentioned in text) on AU565 breast cancer cells at effector to target (E:T) ratios (from 5:1 to 40:1). (**E**,**F**) Comparative cytotoxic efficiency of CTLs following various stimulations at E:T ratio 40:1 on AU565 (HLA-A2^+^/MamA^+^) and MCF7 (HLA-A2^+^/MamA^−^) breast cancer cell lines. (**G**) Cytokines analyzed from the whole cell lysate of CTLs. All experiments were performed in four independent replicates, and data expressed as mean ± SD, *p* < 0.05 compared with MamA2.1 peptide treatment alone. MamA: Mammaglobin-A; ODN: oligodeoxynucleotides.

Previously, we showed that the immunodominant MamA2.1 peptide sequence of the MamA protein induced epitope specific cytotoxic activation of CD8+T lymphocytes (CTLs) in the context of HLA-A2 antigen presentation. To examine the ODN mediated adjuvant potency to further enhance the MamA2.1 specific activation of CTLs, we tested three different ODNs: ODN2216 (class A ODN), ODN2006 (class B ODN), and ODN M362 (class C ODN), along with their respective negative control ODNs (represented by suffix “c” at the end, e.g., ODN 2216c is the negative control for ODN 2216). The adjuvant efficiency of these three ODNs was evaluated by testing the cytotoxic efficiency of CD8+T cells on breast cancer cell lines in the context of HLA-A2 and MamA specificity. Towards this, THP-1 cells (HLA-A2^+^) were pre-treated for 16 hours with various ODNs mentioned above (Figure 1A). Later, we stimulated the naïve CD8+T cells collected from healthy HLA-A2 human subjects for five days with ODNs and MamA2.1-peptide presented by ODN-pretreated THP-1 cells. The activated CD8+T cells (CTLs) were isolated by immunomagnetic beads and tested for their MamA specific cytotoxic functionality by their ability to induce cell lysis of AU565 (HLA-A2^+^/MamA^+^) breast cancer cells for 48 hours. The MCF-7 (HLA-A2^+^/MamA^−^) breast cancer cells were used as negative controls. Initially, we confirmed the MamA2.1 specificity of these newly activated CTLs by MamA2.1-tetramer staining. As shown in Figure 1B,C, activated CTLs following stimulation of naïve CD8+T cells with MamA2.1 peptide presented by THP-1 cells demonstrated significantly enhanced tetramer staining when co-treated with ODN2006 (11.1 ± 1.8%, *p* < 0.05 as compared to the MamA2.1 peptide only) and ODN M362 (12.6 ± 0.9%, *p* < 0.05) as compared to co-treatment with ODN2006 (4.7 ± 0.4%, *p* > 0.05) or peptide alone (4.3 ± 0.7%). These data suggested that ODN2006 and ODN M362 were able to induce up to three-fold higher CTL activation compared to the peptide alone. Next, we confirmed the MamA and the HLA-A2 specific functionality of these newly activated CTLs by their ability to induce cytotoxicity on AU565 and MCF-7 breast cancer cell lines. As shown in Figure 1D–F, newly activated MamA2.1 specific CTLs demonstrated significantly enhanced cytotoxicity on MamA+ and HLA-A2+ AU565 breast cancer cells when co-treated with ODN2006 (69 ± 10%, E:T—40:1, *p* < 0.05 as compared to the MamA2.1 peptide only) and ODN M362 (76 ± 11%, *p* < 0.05) as compared to co-treatment with ODN2006 (27 ± 7%, *p* > 0.05) or peptide alone (28 ± 5%). Importantly, these newly activated CTLs did not exert significant cytotoxicity on MamA-negative MCF-7 breast cancer cells, suggesting that these activated CTLs (either with or without ODNs) were specific to the MamA antigen on breast cancer cells. Furthermore, molecular analysis for the expression of inflammatory and cytotoxic cytokines (TNFα, IFNγ, and granzyme B) in MamA2.1 activated CTLs demonstrated that co-treatment with either ODN2006 or ODN M362 significantly enhanced the expression of these cytokines as compared to co-treatment with ODN2006 or the peptide alone (Figure 1G). Along with this, co-treatment with the respective ODN negative controls (ODN 2006c, M362c, and 2216c) did not lead to significantly higher tetramer staining, cancer cell cytotoxicity, or cytokine expression as compared to the peptide treatment alone, suggesting the specificity of ODN towards MamA2.1 specific CD8+T cell activation. Treatment of naïve CD8+T cells with ODNs alone did not induce enhanced expression of these inflammatory cytokines, suggesting that the MamA specific activation of CTLs was completely mediated by the impact of ODNs exclusively on THP-1 cells’ antigen presentation and co-stimulatory capabilities. Taken together, these data demonstrated that ODN2006 and ODN M362 exert strong adjuvant effects towards MamA specific activation of naïve CD8+T cells to a cytotoxic phenotype. 

### 3.2. ODN2006 and M362 Induced differential Phenotypic Changes in THP-1 Cells

**Figure 2:**
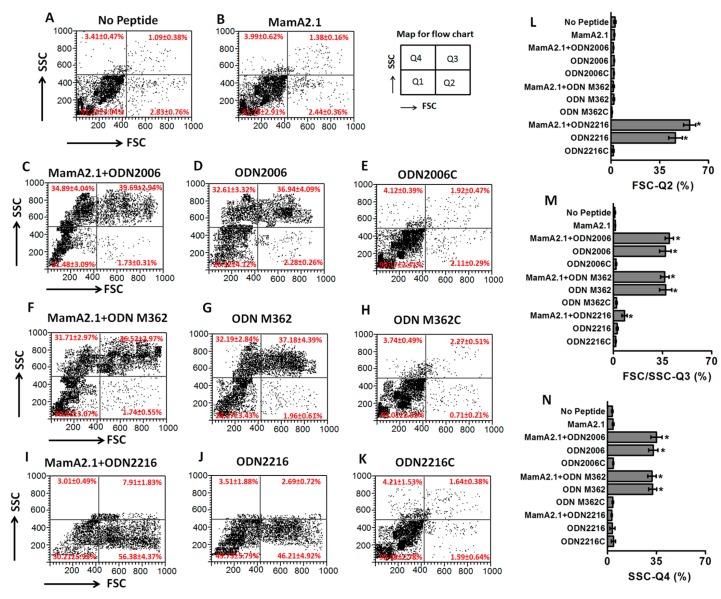
Differential phenotypic changes of THP-1 cells following stimulation with the three ODNs. THP-1 cells were analyzed by flow cytometry under regular culture conditions (**A**), following stimulation with the MamA2.1 peptide (10 µg/mL) (**B**), combination of MamA2.1+ODN2006 (**C**), ODN2006 alone (**D**), negative control for ODN2006—ODN2006c (**E**), combination of MamA2.1+ODN M362 (**F**), ODN M362 alone (**G**), negative control for ODN M362–ODN M362c (**H**), combination of MamA2.1+ODN2216 (**I**), ODN2216 alone (**J**), and negative control for ODN2216–ODN2216c (**K**). Comparative changes in the cell phenotype (as measured by forward scatter, FSC), quadrant-2 (**L**), intracellular complexity (as measured by side scatter, SSC) and cell phenotype, quadrant-3 (**M**), and intracellular complexity alone, quadrant-4 (**N**) are shown. All experiments were performed in four independent replicates, and data expressed as mean ± SD, *p* < 0.05 compared with the MamA2.1 peptide treatment alone.

As our initial studies (mentioned above) suggested that ODNs exerted their effect through modulation of THP-1 functionality, we next verified if ODNs induced unique phenotypic and molecular expression patterns in THP-1 cells. Importantly, as ODNs are considered to induce innate immune responses, we also wanted to study the differential impact of ODN2006 and ODN M362 versus ODN2216 on THP-1 cells. Towards this, we performed flow cytometry-based studies to determine the cell size [as evidenced by forward scatter (FSC)] and changes in the complexity or granular density [as evidenced by side scatter (SSC)] of THP-1 cells following peptide and ODN treatment (either combined or alone). As shown in Figure 2A,B, the MamA2.1 peptide treatment alone did not induce any morphological or granular changes in the THP-1 cells. Following treatment with ODN2006 (Figure 2C–E), 37% (*p* < 0.05) of the THP-1 cells demonstrated an enhanced FSC and SSC as compared to 1.4% with the peptide treatment alone (Figure 2B) in this quadrant. Furthermore, upon treatment with ODN2006, 33% (*p* < 0.05) of the THP-1 cells demonstrated an enhanced SSC only as compared to 4% with the peptide treatment alone in this quadrant. There was no significant difference in the morphological or the granular features of THP-1 cells between the combined treatment with the MamA2.1 peptide and ODN2006 (Figure 2C) versus ODN2006 alone (Figure 2D). The negative control for ODN2006, the ODN2006c, did not induce any morphological changes in THP-1 cells. Similar to ODN 2006, the ODN M362 (Figure 2F–H) also induced significant phenotypic changes in the THP-1 cells. However, it is interesting to note that ODN2216 treatment (Figure 2I–K) induced a significant shift in the FSC alone (46%, *p* < 0.05, compared to MamA2.1 peptide treatment alone), with no impact on the SSC and combined FSC and SSC. These data in Figure 2 clearly suggest that ODN2006 and M362 induced morphological changes (FSC) and potentially enhanced expression of cytokines, leading to their effect towards the activation of CTLs (Figure 1).

**Figure 3:**
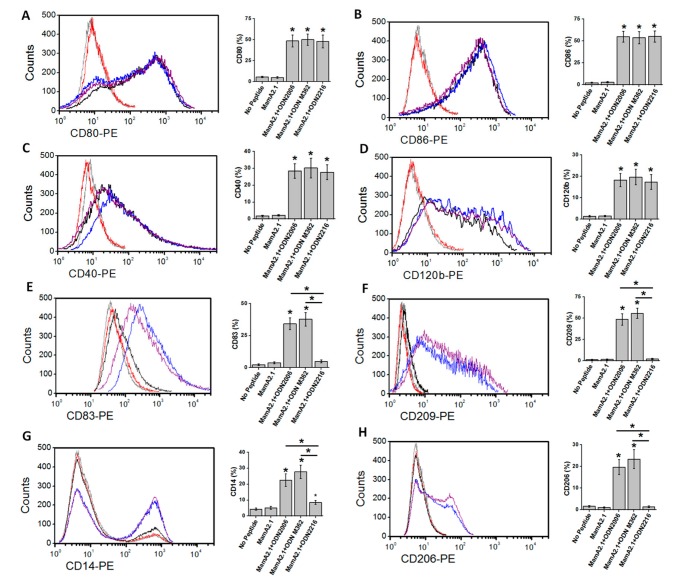
Expression of phenotypic markers on THP-1 cells following treatment with ODNs. The various treatment conditions were: (light gray) no peptide—regular culture conditions; (red) MamA2.1 peptide alone; (magenta) MamA2.1+ODN2006; (blue) MamA2.1+ODN M362; and (black) MamA2.1+ODN2216. All three ODNs induced an enhanced expression of cell maturation markers, namely CD80 (**A**), CD86 (**B**), CD40 (**C**), and CD120b (**D**). However, only ODN2006 and M362 induced enhanced expression of phagocytic markers, namely CD83 (**E**), CD209 (**F**), CD14 (**G**), and CD206 (**H**). All experiments were performed in four independent replicates, and data are expressed as mean ± SD, * *p* < 0.05 compared with the MamA2.1 peptide treatment alone.

As activation of CTLs by THP-1 is dependent on the antigen presentation capability of THP-1 cells, we next tested if morphological changes in THP-1 (Figure 2) following treatment with ODN2006 and M362 are associated with the molecular profile specific to enhanced phagocytic dendritic-cell-like differentiation. Towards this, we verified the expression of cell maturation markers (CD80, CD86, CD40, and CD120b) and phagocytic markers (CD83, CD209, CD14, and CD206) [26,27,28]. As shown in Figure 3A–D, all ODNs were able to induce enhanced expression of macrophage-like maturation markers, namely CD80, CD86, CD40, and CD120b. However, only ODN2006 and M362 (Figure 3E–H) induced enhanced expression of phagocytic markers, namely CD83, CD209, CD14, and CD206. These data suggest that ODN2006 and M362 caused enhanced phagocytic dendritic-cell-like differentiation of THP-1 cells, leading to the enhanced antigen presentation capability of CD8+T-cells and subsequently resulting in boosted cytotoxic functionality of these CTLs on breast cancer cells.

We performed a detailed LUMINEX-based cytokine analysis on THP-1 cells following ODN treatment. As shown in Table 1, ODN2006 and M362 (and not ODN2216) treatment to THP-1 cells specifically enhanced the expression of immunostimulatory cytokine IL-12p70 and chemoattractant cytokines MCP1, MIP1α, and MIP1β. Meanwhile, expression of other inflammatory cytokines, such as IL-6, RANTES, TNFα, IFNγ, IL-1β, and vascular endothelial growth factor (VEGF) was increased by treatments with all three ODNs to THP-1 cells. These cytokine data (Table 1) correlated with our phenotypic data (Figure 2) to demonstrate that ODN2006 and M362 specifically induced expression of the macrophage co-stimulatory molecule (IL-12), which plays a critical role in the MamA2.1 peptide specific activation of CD8+T cells [29]. Taken together, all these data suggest that ODN2006 and M362 exerted their adjuvant capability by specific enhancement of antigen presentation and co-stimulatory capability towards the activation of naïve CD8+T cells to the MamA antigen specific cytotoxic CTLs.

### 3.3. Upregulation of TLR-9/6 Pathways in THP-1 Cells following Co-Treatment with ODN2006 and M362

**Figure 4:**
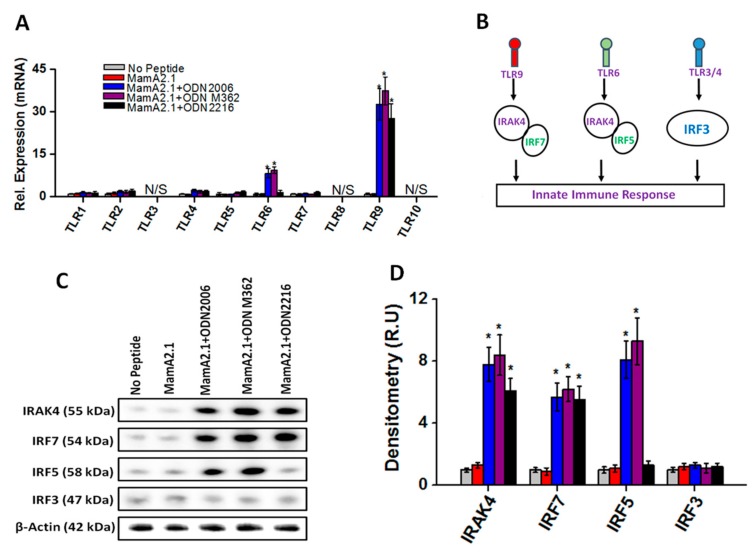
Enhanced expression of TLR-6 and -9 on THP-1 cells following treatment with ODNs. (**A**) Relative mRNA expression of TLRs in THP-1 cells following various treatment conditions mentioned above. (**B**) Schematic of the TLR mediated innate inflammatory response. (**C**,**D**) Protein expression and densitometry analysis of TLR mediated signaling factors, namely IRAK4, IRF7, IRF5, and IRF3. All experiments were performed in four independent replicates, and data are expressed as mean ± SD, * *p* < 0.05 compared with the MamA2.1 peptide treatment alone.

As ODNs are known to induce TLR mediated innate immune responses, we next determined if there is any change in TLR mediated signaling following treatment with various ODNs. As shown in Figure 4A, co-treatment of the MamA2.1 peptide with either ODN2006 or M362 demonstrated enhanced mRNA levels of TLR-9 and -6, while other TLRs- (1–11 except 6 and 9) did not demonstrate any significant change in the expression pattern under similar conditions. Further co-treatment with ODN2216 demonstrated enhanced mRNA levels of TLR-9 with no significant change in TLR-6. It is also important to note that treatment with the MamA2.1 peptide alone did not alter the expression level of TLR-6 and -9, suggesting that the enhanced expression of TLR-9/6 was not peptide-dependent but was rather dependent upon treatment with ODN2006 or M362. Literature evidence has shown TLR-9 induces down-stream signaling through IRAK4/IRF7 factors, TLR-6 induces down-stream signaling through IRAK4/IRF5 factors, and TLR-3/4 induces down-stream signaling through the IRF3 molecule (Figure 4B) [30]. Western blot analysis (Figure 4C,D) demonstrated that, following co-treatment of the MamA2.1 peptide with ODN2006 or M362, there was enhanced protein expression of IRAK4, IRF7, and IRF5, while co-treatment of the MamA2.1 peptide with ODN2216 induced enhanced protein expression of IRAK4 and IRF7 only. However, the expression pattern of IRF3 remained unchanged under treatment conditions. These data suggest that ODN2006 and M362 exerted their downstream signaling in THP-1 cells through TLR-9/6 pathways, while ODN2216 induced only the TLR-9 pathway. 

**Figure 5:**
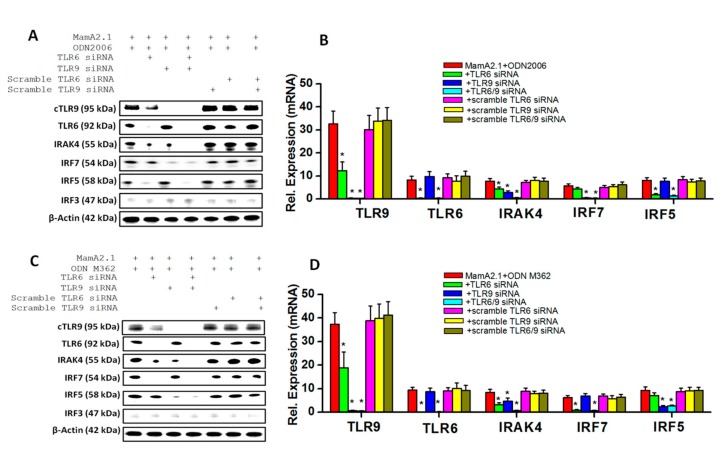

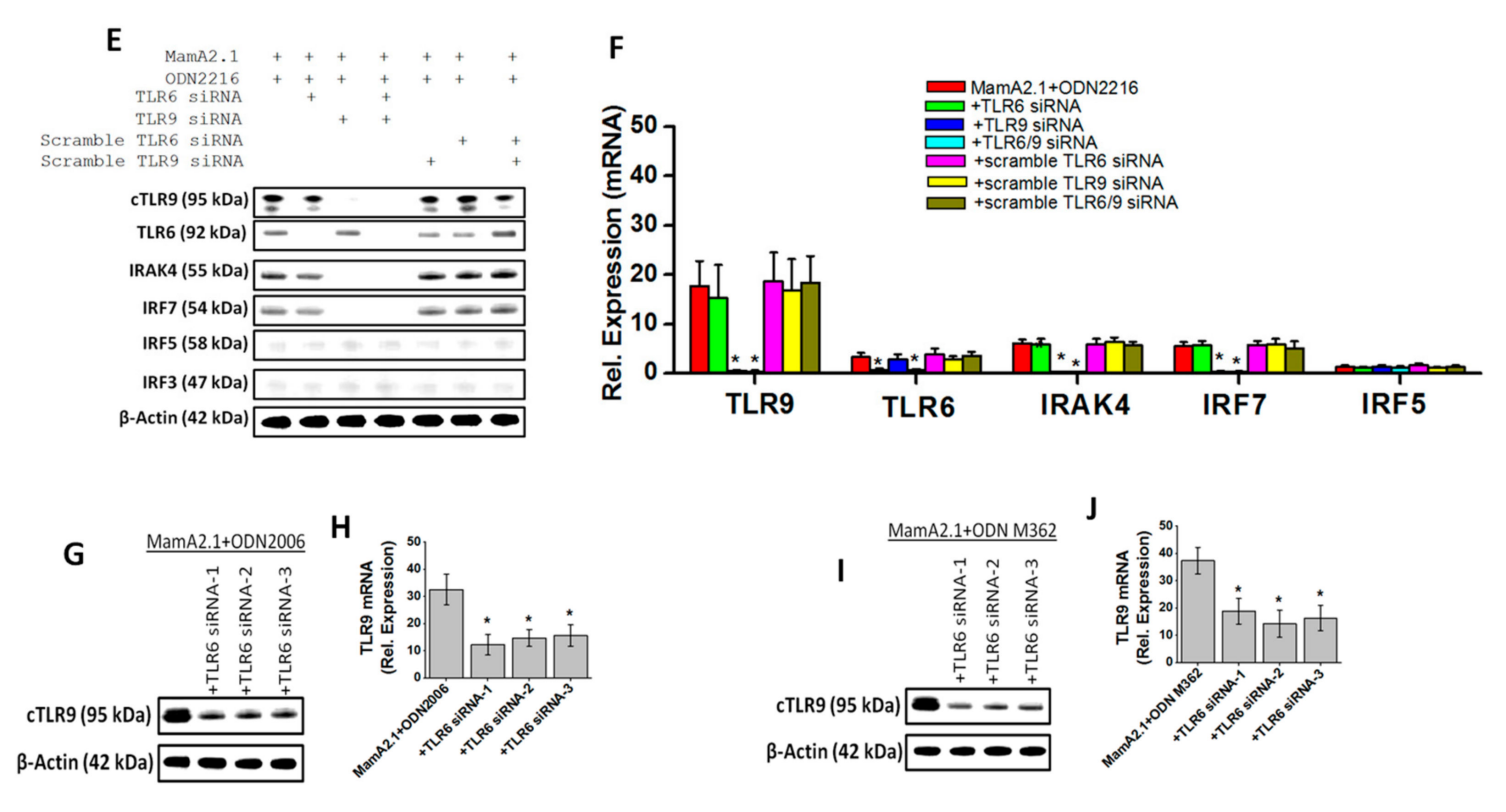
Interplay of TLR-6/-9 signaling in THP-1 cells following treatment with ODN2006 and M362. Protein expression and densitometry analysis of TLR-6/-9 proteins and their downstream signaling factors, namely IRAK4, IRF7, IRF5, and IRF3, following treatment with ODN2006 (**A**,**B**), ODN M362 (**C**,**D**), and ODN2216 (**E**,**F**). As TLR-6 siRNA treatment induced decreased expression of TLR-9 (**A**–**D**), we checked if this could be due to off-target effects. However, as shown in (**G**–**J**), three different TLR-6 siRNAs induced the decreased expression of TLR-9, suggesting interplay between TLR-6 and -9 expression. All experiments were performed in four independent replicates, and data are expressed as mean ± SD, * *p* < 0.05 compared with the MamA2.1 peptide treatment alone.

To confirm these signaling pathways (mentioned above), we utilized siRNA-based knock down of TLR-9 and TLR-6 following co-treatment with ODNs. As shown in Figure 5A,B, knock down of TLR-9 along with co-treatment with the MamA2.1 peptide and ODN2006 demonstrated a decreased expression of IRF7, and knock down of TLR-6 under similar conditions demonstrated decreased expression of IRF5. As expected, IRAK-4 demonstrated what was apparently an additive decreased expression following knock down of TLR-9 and -6 combined together. These data suggest that ODN2006 was able to induce both TLR-9 and -6 signaling. Similar effects of enhanced TLR-9/-6 signaling was noticed following treatment with ODN M362 (Figure 5C,D). However, knock down of TLR-9 following co-treatment of ODN2216 with the MamA2.1 peptide (Figure 5E,F) demonstrated decreased expression of IRAK4 and IRF7, and no change in expression of IRAK4 or IRF5 was noticed following knock down of TLR-6 under similar treatment conditions. Taken together, these data suggest that ODN2006 and ODN M362 demonstrated enhanced vaccine adjuvant effects through the upregulation of both TLR-9 and -6 signaling, while ODN2216 was able to induce only TLR-9 signaling. It is interesting to note that the siRNA-based knock down of TLR-6 reduced the protein expression of TLR-9. To check if this could be the off-target effect of siRNA, we repeated our studies with three different TLR-6 siRNA, and all three showed similar results (Figure 5G–J). However, knock down of TLR-9 did not show any change in TLR-6 expression. These data suggest an interdependence of TLR-9/-6 signaling, which was explored further in our study.

**Figure 6:**
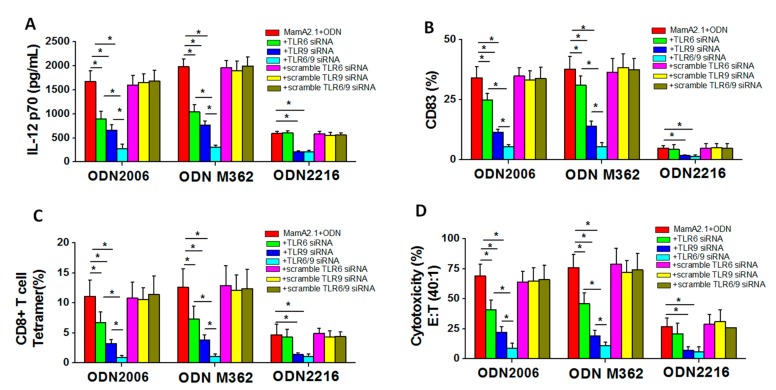
Expression of the cytokine of phenotypic differentiation markers following TLR-6 and/or -9 knock down along with co-treatment of the MamA2.1 peptide and various ODNs. The IL-12 cytokine release (**A**) and the expression maturation marker CD83 (**B**) in THP-1 cells following various treatment conditions. The ability of these stimulated THP-1 cells to induce cytotoxic CD8+T cell responses was studied by tetramer staining (**C**) and cytotoxicity on AU565 breast cancer cells (**D**). All experiments were performed in four independent replicates, and data are expressed as mean ± SD, * *p* < 0.05 compared with the MamA2.1 peptide treatment alone.

To determine if TLR-9/-6 signaling played a direct downstream impact on the functionality of THP-1 cells following co-treatment with the MamA2.1 peptide and ODN2006 or M362, we checked for the IL-12 cytokine release (Table 1) and the expression maturation marker CD83 (Figure 3). We also checked for the ability of these THP-1 cells to induce cytotoxic CD8+T cell responses by testing for tetramer staining and cytotoxicity on breast cancer cells (Figure 1). As shown in Figure 6A, knock down of TLR-9 and/or -6 following co-treatment of THP-1 cells with either ODN2006 or M362 demonstrated an apparent cumulative effect towards the inhibition of macrophage-specific IL-12p70 cytokine release. However, when THP-1 cells were co-treated with ODN2216, only knock down with TLR-9 demonstrated a significant decrease in the secretion of the IL-12p70 cytokine, and TLR-6 knock down had no effect on IL-12p70 release. We next tested for the expression of the CD83 dendritic cell-like maturation marker following treatment with ODNs on THP-1 cells. As shown in Figure 6B, knock down of TLR-9 and/or -6 following co-treatment of THP-1 cells with either ODN2006 or M362 demonstrated an apparent cumulative effect towards the inhibition of the expression of the CD83 molecule. However, when THP-1 cells were co-treated with ODN2216, only knock down with TLR-9 demonstrated a significant decrease in the expression of the CD83 molecule, and TLR-6 knock down had no effect on CD83 expression. As ODN2006 and M362 treatment demonstrated an enhanced dendritic-cell-like maturation of THP-1 cells, we tested the role of TLR-9/-6 signaling on the impact of co-treated THP-1 cells towards the induction of CD8+T cell immune responses. As shown in Figure 6C, co-culture of CD8+T cells isolated from HLA-A2+ donor with the MamA 2.1 peptide and THP-1 cells pretreated with ODN2006 and the MamA2.1 peptide along with TLR-9 and/or -6 knockdown demonstrated an apparent cumulative inhibition of MamA2.1 tetramer staining. Similar results (as ODN2006 treatment) were obtained from CD8+T cells following co-treatment with ODN M362 under treatment conditions. However, upon ODN2216 treatment conditions, the MamA2.1 tetramer staining of CD8+T cells did not show any impact following co-culture with TLR-6 knock down THP-1 cells, while there was decreased tetramer staining in CD8+T cells following co-culture with TLR-9 knock down THP-1 cells under similar conditions. Cytotoxicity of the activated CD8+T cells on AU565 (HLA-A2+MamA+) breast cancer cells yielded similar results as noted above. Taken together, these data suggest that ODN2006 and M362 specifically induced a more phagocytic dendritic-cell-like maturation of THP-1 cells through the synergistic upregulation of TLR-9/-6 signaling pathways, leading to enhanced HLA class-I mediated activation of CD8+T cells and resulting in vaccine-mediated cytotoxicity on breast cancer cells.

### 3.4. Critical Role of IL-12 in Synergistic Effect of TLR-9/-6 Signaling following Treatment with ODN2006 and M362

**Figure 7:**
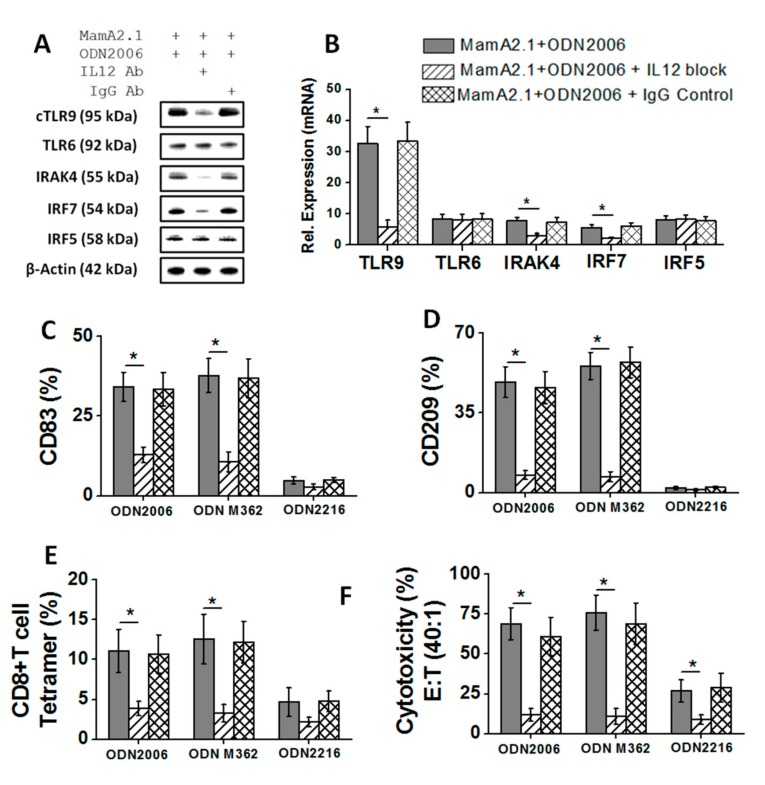
TLR-6/-9 induced IL-12 secretion mediated the auto-stimulated enhanced expression of TLR-9. (**A**,**B**) Protein expression and densitometry analysis of TLR-6/-9 proteins and their downstream signaling factors, namely IRAK4, IRF7, IRF5, and IRF3, following IL-12 blocking by specific monoclonal antibodies with co-treatment of MamA2.1+ ODN2006. IL-12 cytokine release (**C**) and expression maturation marker CD83 (**D**) in THP-1 cells following IL-12 blocking under these treatment conditions. The ability of IL-12 blocked and ODN2006 stimulated THP-1 cells to induce cytotoxic CD8+T cell responses was studied by tetramer staining (**E**) and cytotoxicity on AU565 breast cancer cells (**F**). All experiments were performed in four independent replicates, and data are expressed as mean ± SD, * *p* < 0.05 compared with the MamA2.1 peptide treatment alone.

We encountered a puzzling question during our studies (Figure 4, Figure 5 and Figure 6)—if ODNs are known ligands for TLRs, why did the expression of TLR-9 change (increase) following treatment with TLR ligands? As IL-12 is known to play a critical role in macrophage activation [31,32,33], we tested a hypothesis that upregulation of IL-12 secretion following ODN2006 and M362 treatment would exert a positive feedback role toward the enhanced expression of TLRs, thus amplifying the impact of these ODNs. As mentioned above, TLR-9/-6 exerted a synergistic effect towards increased IL-12p70 secretion following co-treatment with ODN2006. We blocked the IL-12 auto-stimulation on THP-1 cells through blocking with the IL-12p79 specific monoclonal Abs. As shown in Figure 7A,B, addition of IL-12 Abs to THP-1 cell cultures following co-treatment with ODN2006 and the MamA2.1 peptide demonstrated a decreased expression of TLR-9 with no impact on TLR-6 expression. This effect was also associated with decreased expression of TLR-9 specific signaling factors, IRAK4 and IRF-7, with no impact on IRF-5, a TLR-6 specific signaling factor. These data suggested that IL-12 induced an auto-stimulation effect on TLR-9 and caused the enhanced expression and the eventual TLR-9 mediated downstream signaling. To further evaluate the role of IL-12 inducing the functionality changes in THP-1 cells following co-treatment with ODN2006 and the MamaA2.1 peptide, we tested for the expression of dendritic-cell-like maturation markers, CD83 and CD209. As shown in Figure 7C,D, addition of IL-12 Abs to THP-1 cell cultures following co-treatment with ODN2006 and the MamA2.1 peptide demonstrated a decreased expression of CD83 and CD209, suggesting that IL-12 auto-stimulation induced an amplifying effect on the THP-1 maturation to dendritic-cell-like phagocytic functionality. We finally evaluated the impact of IL-12 on the induction of CD8+T cells by pre-ODN-treated THP-1 cells by the above mentioned procedure. As shown in Figure 7E,F, a co-culture of CD8+T cells isolated from the HLA-A2+ donor with the MamA 2.1 peptide and the THP-1 cells pretreated with ODN2006, the MamA2.1 peptide, and IL-12 Abs demonstrated an inhibition of MamA2.1 tetramer staining and CD8+T cell mediated cytotoxicity on AU565 breast cancer cells. Taken together, these data point towards a critical role of IL-12 mediating an auto-stimulatory effect to enhance the TLR-9 signaling mechanism following co-treatment of ODN2006 with the MamA2.1 peptide on THP-1 cells.

### 3.5. In Vivo Reduction in Tumor Progression following Adoptive Transfer of CD8+T Lymphocytes from MamA2.1 Peptide Vaccinated Mice with Co-Treatment of ODN2006 and M362

**Figure 8:**
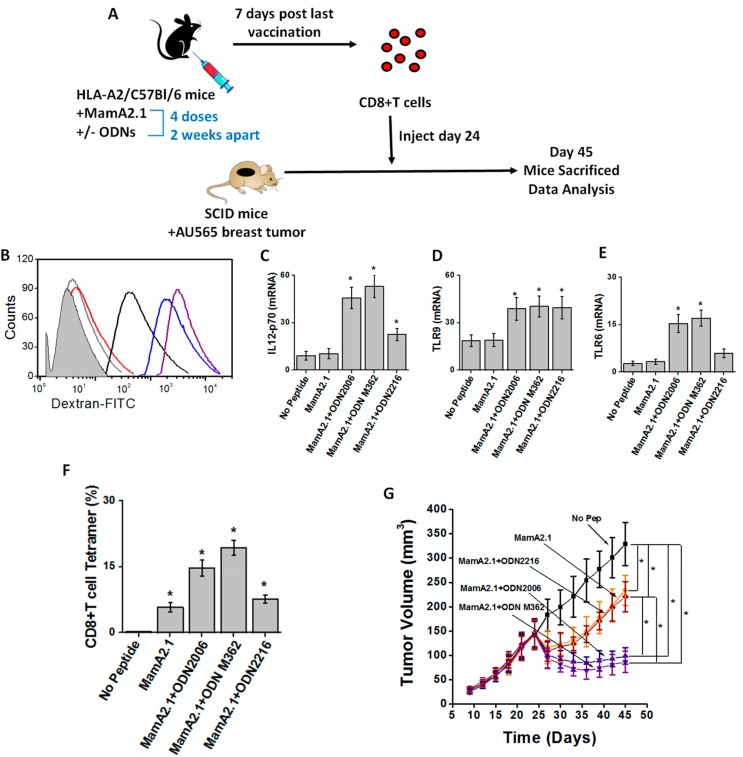
In vivo reduction in tumor progression following adoptive transfer of CD8+T lymphocytes from the MamA2.1 peptide vaccinated mice with co-treatment of ODN2006 and M362. (**A**) Schematic of the murine tumor experiments. (**B**) Dextran uptake assay to determine phagocytic activity of the peritoneal macrophages from HLA-A2+/C57Bl/6 mice co-treated with the MamA2.1 peptide vaccination with and without the three ODNs; (light gray) no peptide—regular culture conditions; (red) MamA2.1 peptide alone; (magenta) MamA2.1+ODN2006; (blue) MamA2.1+ODN M362; and (black) MamA2.1+ODN2216. IL-12 cytokine release (**C**), expression of TLR-6 (**D**) and -9 (**E**) in peritoneal macrophages, and tetramer staining for MamA2.1 in CD8+T cells collected from spleens (**F**) obtained from mice following the various vaccination conditions mentioned above. (**G**) Tumor progression in severe combined immunodeficient (SCID)/beige mice injected with AU565 breast cancer cells followed by the adoptive transfer of CD8+T cells obtained from HLA-A2+/C57Bl/6 mice treated with peptide vaccination under various conditions, as mentioned above. All experiments were performed in *n* = 5, and data are expressed as mean ± SEM (standard error of mean). Statistical significance was determined by Wilcoxon nonparametric tests. Differences in graft survival were assessed by means of two-way log-rank (Mantel-Haenszel) tests. Significance was set a-priori at * *p* < 0.05.

To study the adjuvant effect of ODNs under in vivo conditions, we utilized murine breast tumor models. As shown in Figure 8A, we first vaccinated intraperitoneally into HLA-A2/C57bl/6 transgenic mice with the MamA2.1 peptide with or without one of the three ODNs (ODN2006, M362, or 2216). Four doses of this vaccination were administered in two-week intervals between each dose. Seven days following the last dose, CD8+T cells were isolated from the spleen of these mice and injected into SCID/beige mice with 24-day old AU565 orthotopically implanted breast tumors. The peritoneal macrophages isolated from vaccinated HLA-A2+/C57Bl/6 transgenic mice demonstrated enhanced phagocytic functionally, as shown by dextran uptake assay (Figure 8B), IL-12p70 secretion (Figure 8C), and expression of the innate immune markers TLR-9 (Figure 8D) and TLR-6 (Figure 8E) following co-vaccination of the MamA2.1 peptide with either ODN2006 or M362. These data were in good alignment with our in vitro studies mentioned above. Further, adoptive transfer of CD8+T cells from transgenic mice vaccinated with the MamA2.1 peptide and either ODN2006 or M362 demonstrated significantly reduced tumor growth compared to CD8+T cells transferred from the MamA2.1 peptide alone or with vaccination or co-vaccination with the MamA2.1 peptide and ODN2216. Similarly, we noticed an enhanced MamA2.1 tetramer staining on CD8+T cells isolated from mice co-vaccinated with the MamA2.1 peptide and either ODN2006 or M362. Taken together, all these data strongly demonstrate that ODN2006 and ODN M362 exert a strong adjuvant effect on MamA-based peptide vaccination, leading to diminished MamA expressing tumor growth.

## 4. Discussion

Vaccine adjuvants play a critical role in enhancing the ability of the host to mount inflammation and innate immune responses to accentuate the antigen specific adaptive immune responses. Adjuvants could also reduce the number of doses and/or the frequency of vaccine administration needed to achieve the best immunological outcome. The current understanding suggests that vaccine adjuvants—through the upregulation of non-specific innate immune mediated inflammatory cascades at the site of the vaccine application—leads to the activation of the co-stimulatory signaling needed to induce long-term viable antigen specific CD8+T lymphocyte effectors and memory responses [34].

Multiple preclinical studies have established that ODNs have strong vaccine adjuvant functionality. To date, several unique classes of ODNs have been recognized based on the structure–function impact on the immune system. Although all unique classes of ODNs have unmethylated CpG motifs containing single stranded oligodeoxynucleotides, they differ in structure and immunological activity. The class A ODNs (e.g., ODN2216) have phosphodiester bonds in core nucleotides with phosphorothioate bonds in terminal nucleotides. They have a single CpG motif flanked by palindromic sequences, resulting in a stem-loop structure. They are generally considered mild with limited innate and inflammatory activation [35]. The class B ODNs (e.g., ODN2006) contain one to five CpG motifs with complete phosphorothioate backbones, making them resistant to nuclease damage. Class B ODNs have a half-life of 30–60 min in intracellular and biological microenvironments as compared to 5–10 min for class A ODNs. These ODNs trigger efficient inflammatory responses in antigen presenting cells and humoral immune cells [36]. As with the class B ODNs, the class C ODNs (e.g., ODN M362) have phosphorothioate nucleotide backbones. However, as with the class A ODNs, they also have palindromic motifs to form stem-loop structures or dimers. The class C ODNs are considered to potentiate cytokine release, resulting in enhanced cellular and humoral immune responses [37]. Interestingly, our current data (Figure 1) demonstrated that class B and C ODNs (ODN2006 and M362, respectively) induced higher CTL activation compared to class A ODNs (ODN2216).

Activation of TLR by an appropriate ligand leads to the upregulation of the production of pro-inflammatory cytokine cascades—specifically, interferon (IFN) responses. One of these innate immune system molecules, TLR9, is well-known to specifically recognize and interact with bacterial specific unmethylated CpG motifs to initiate an immune response [38]. It is also important to note that, upon interaction of TLR9 with ODN, there is a downstream signaling cascade mediated by several cytoplasmic and nuclear signaling factors. These signaling events proceed through the recruitment of MyD88 (myeloid differentiation factor 88), IRAK (interleukin receptor associated kinase), TRAF6 (tumor necrosis factor receptor-associated factor 6), and MAPK (mitogen-activated kinases), culminating in the secretion of pro-inflammatory chemokines and cytokines [39]. A phase III/IV clinical study by Fourcade et al. on melanoma patients demonstrated that the addition of CpG ODN as an adjuvant to a vaccination with NY-ESO1, a melanoma specific antigen, significantly increased the antigen specific CTL frequency in the peripheral blood of these melanoma patients, suggesting ODN plays a critical role in exaggerating the vaccine immune response [40]. Furthermore, it is interesting to note that ODN not only enhanced the CD8+T cell responses but was also effective in priming antigen specific CD4+T cell and humoral immune responses [41]. Our current data are in line with these previous studies, as we demonstrated that class B and C ODNs (ODN2006 and ODN M362, respectively) induced higher TLR9 expression compared to class A ODNs (ODN2216). Furthermore, siRNA mediated knock down of TLR9 significantly abrogated CTL activation. These studies suggest that ODNs (2006 and M362) act as potent adjuvants to further exaggerate the CD8+T cells immunogenicity of MamA based DNA or peptide vaccines. Future studies are warranted to study the impact of ODN2006 and M362 adjuvant applications towards the induction of CD4+T cell and humoral immune responses. Furthermore, dot-plot analyses of our flow cytometry data (Figure 2) suggested that ODN-mediated activation of THP-1 cells induced multiple subtypes of dendritic-cell-like phenotypes. This requires more detailed future studies with isolation of various cell subtypes and multi-color (such as 14-color) flow cytometry-based experiments to characterize the functionality of these distinct subsets of dendritic-cell-like phenotype differentiation by ODNs.

**Figure 9:**
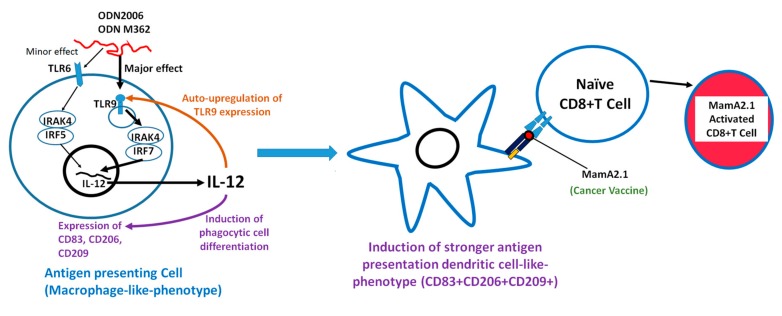
Possible mechanism of ODN2006 and M362 mediated TLR-6/9 interplay mediated by IL-12 leading to THP-1 cell differentiation and phagocytic capability, resulting in antigen specific CTL activation.

We thought it important to determine the possible reason for the enhanced expression of TLR-9 following treatment with its ligands. To this end, we performed IL-12 blocking studies, which demonstrated that antagonizing this cytokine decreased the expression of TLR-9. This suggested that IL-12 expression had an auto-stimulatory effect in the synergistic effect of TLR-6/9 induced expression of IL-12, and this cytokine consequently enhanced the expression of the TLR-9 protein (Figure 9). Our cytokine data (Table 1) also showed that there was an enhanced VEGF expression in THP-1 cells following ODN2006 and M362 treatment. VEGF is known to induce angiogenesis and treatment resistance in patients. However, future studies are needed to study the efficiency of ODNs in MamA-based DNA vaccines in human clinical trials, as is a long term follow-up to check for the possible development of VEGF-mediated tumor resistance. Furthermore, other adjuvant strategies to enhance class I HLA expression to enhance CTL activation would greatly enhance the future success of the MamA DNA vaccine. Although our current study is limited to the HLA-A2 restricted immunodominant sequence of MamA, future studies testing for the immunodominant sequences of other class I HLA molecules, such as A3, B7, A24, etc., will be needed to determine the broader application of the MamA-based vaccine on the human cancer patient population.

## 5. Conclusions

Our data strongly suggest that ODN-based adjuvants significantly enhance the success of MamA based vaccination strategies. Specifically, we demonstrated that ODN2006 and ODN M362 exerted a strong adjuvant effect through the induction of the initial innate immune response through TLR-9 upregulation, leading to subsequent enhanced MamA specific CTL mediated adaptive immune responses. Therefore, ODN2006 and M362 may provide new venues of application as MamA vaccine adjuvants for better outcomes for breast cancer patients.

## Figures and Tables

**Table 1:** Expression of cytokines/chemokines in THP-1 cells following treatment with various ODNs. All experiments were performed in four independent replicates, and protein values are presented as mean ± SD. * *p* < 0.05 (ANOVA, one-way) compared with the MamA2.1 peptide treatment alone.

Cytokines/Chemokines	No Pep	MamA2.1	MamA2.1 + ODN2006	MamA2.1 + ODN M362	MamA2.1 + ODN2216
**Immunostimulatory Cytokines**					
IL-2	5.2 ± 0.9	6.1 ± 1.2	13.6 ± 2.7	17.2 ± 6.4	7.7 ± 1.4
IL-5	n.d.	n.d.	4.9 ± 1.4	6.3 ± 1.9	n.d.
IL-6	157 ± 27	148 ± 23	294 ± 62 *	342 ± 78 *	311 ± 57 *
IL-7	6.1 ± 1.7	5.3 ± 2.2	10.9 ± 3.3	7.2 ± 2.7	6.8 ± 1.3
IL-8	7651 ± 918	8143 ± 1126	13278 ± 1989	14598 ± 1839	10367 ± 772
IL-12p70	163 ± 43	182 ± 31	1672 ± 231 *	1981 ± 161 *	593 ± 49 *
IL-13	2.9 ± 0.6	3.3 ± 1.1	11.9 ± 2.4	14.7 ± 4.3	4.8 ± 1.3
IL-15	n.d.	n.d.	7.2 ± 1.9	9.3 ± 3.7	n.d.
Eotaxin	47 ± 16	34 ± 9	59 ± 17	52 ± 18	41 ± 9
IP-10	12 ± 4	14 ± 5	31 ± 9	27 ± 9	21 ± 7
**Anti-inflammatory Cytokines**					
IL-4	n.d.	n.d.	n.d.	2.9 ± 0.6	n.d.
IL-10	42 ± 12	37 ± 12	19 ± 6	14 ± 6	33 ± 12
IL-1RA	2981 ± 343	2719 ± 469	1516 ± 121 *	1673 ± 131 *	2528 ± 154
**Chemoattractants**					
MCP-1	390 ± 56	422 ± 109	736 ± 152	787 ± 172	496 ± 101
MIP-1α	312 ± 121	431 ± 76	784 ± 112	856 ± 143	533 ± 163
MIP-1β	665 ± 116	726 ± 91	1167 ± 128	1218 ± 109	881 ± 179
RANTES	2139 ± 158	2432 ± 338	7819 ± 911 *	8337 ± 763 *	7917 ± 967 *
**Effector Cytokines**					
TNF-α	598 ± 72	538 ± 69	3871 ± 403 *	5178 ± 476 *	1267 ± 391 *
IFN-γ	127 ± 19	146 ± 21	449 ± 127	503 ± 93	312 ± 72
IL-1β	1439 ± 96	1235 ± 108	1922 ± 216	1822 ± 177	1432 ± 876
IL-17	11 ± 3	14 ± 4	19 ± 4	15 ± 4	16 ± 5
**Growth Factors/Cell differentiation factors**					
IL-9	n.d.	n.d.	6.4 ± 2.1	6.1 ± 1.7	3.2 ± 0.8
FGF-basic	n.d.	n.d.	n.d.	n.d.	n.d.
PDGF-BB	23 ± 8	19 ± 3	27 ± 7	24 ± 8	31 ± 6
VEGF	3415 ± 482	2769 ± 511	5853 ± 721 *	7241 ± 818 *	7471 ± 917 *
G-CSF	456 ± 89	514 ± 132	738 ± 171	674 ± 133	598 ± 123
GM-CSF	31 ± 9	36 ± 8	53 ± 14	44 ± 12	54 ± 16
